# Assessment of Motor Function, Sensory Motor Gating and Recognition Memory in a Novel BACHD Transgenic Rat Model for Huntington Disease

**DOI:** 10.1371/journal.pone.0068584

**Published:** 2013-07-11

**Authors:** Yah-se K. Abada, Huu Phuc Nguyen, Rudy Schreiber, Bart Ellenbroek

**Affiliations:** 1 Neuropharmacology, EVOTEC AG, Hamburg, Germany; 2 Brain Research Institute Department of Neuropharmacology, University of Bremen, Bremen, Germany; 3 Institute of Medical Genetics and Applied Genomics, University of Tübingen, Tübingen, Germany; 4 Behavioral Physiology & Pharmacology, University of Groningen, Groningen, The Netherlands; 5 School of Psychology, Victoria University of Wellington, Wellington, New Zealand; National Center of Neurology and Psychiatry, Japan

## Abstract

**Rationale:**

Huntington disease (HD) is frequently first diagnosed by the appearance of motor symptoms; the diagnosis is subsequently confirmed by the presence of expanded CAG repeats (> 35) in the *HUNTINGTIN* (*HTT*) gene. A BACHD rat model for HD carrying the human full length mutated *HTT* with 97 CAG-CAA repeats has been established recently. Behavioral phenotyping of BACHD rats will help to determine the validity of this model and its potential use in preclinical drug discovery studies.

**Objectives:**

The present study seeks to characterize the progressive emergence of motor, sensorimotor and cognitive deficits in BACHD rats.

**Materials and Methods:**

Wild type and transgenic rats were tested from 1 till 12 months of age. Motor tests were selected to measure spontaneous locomotor activity (open field) and gait coordination. Sensorimotor gating was assessed in acoustic startle response paradigms and recognition memory was evaluated in an object recognition test.

**Results:**

Transgenic rats showed *hyper*activity at 1 month and *hypo*activity starting at 4 months of age. Motor coordination imbalance in a Rotarod test was present at 2 months and gait abnormalities were seen in a Catwalk test at 12 months. Subtle sensorimotor changes were observed, whereas object recognition was unimpaired in BACHD rats up to 12 months of age.

**Conclusion:**

The current BACHD rat model recapitulates certain symptoms from HD patients, especially the marked motor deficits. A subtle neuropsychological phenotype was found and further studies are needed to fully address the sensorimotor phenotype and the potential use of BACHD rats for drug discovery purposes.

## Introduction

Huntington disease (HD) is a progressive neurodegenerative disorder that is associated with widespread degeneration of cortical neurons and striatal medium spiny neurons (MSN) [1–3]. The neuronal loss is caused by an expanded polyglutamine tract (> 35 CAG) in the *HUNTINGTIN* (*HTT*) gene on chromosome 4 [4]. Huntingtin (htt) has many functions in cells and is essential for development [5–8]. It is not well-known why MSN neurons are selectively vulnerable in HD [9]. A wide variety of motor, cognitive and neuropsychiatric symptoms have been observed in HD patients [10,11]. As the disease progresses, patients become completely dependent and eventually require full-time care. To date, clinically proven treatments that can cure or halt the disease’s progression have not yet been discovered.

Important insights into the pathogenic mechanisms in HD were gained by the development and use of multiple transgenic murine models. At present, a number of knock-in and transgenic mouse models, expressing the N-terminal fragment of HD exon 1 (e.g. R6/2 mice [12]) or the full-length endogenous / human mutant htt (such as the BACHD mice [13]) provide ample opportunities to study the chronically progressing phenotype of the disease [14]. A good animal model should reflect as many of the neuropathological and clinical symptoms of the human disease as possible [15]. The BACHD mouse model of HD, for example, recapitulates several aspects of the neuropathology and symptoms, including formation of mutant htt positive aggregates, reduction in cortical BDNF mRNA expression levels, cognitive, emotional, motor and sensory gating deficits [13,16–20]. Notwithstanding the value of existing mouse HD models, certain behavioral processes related to learning and memory and pharmacological validation are typically more challenging to evaluate in mice [21]. This is one of the reasons why the availability of HD models in other species is important. Certain aspects of the behavioral repertoire of rats are more adequate and/or accessible for assessment in specific tasks, such as in learning and memory models. A BACHD rat model of HD has recently been established. This rat model is of particular interest since it expresses, like the BACHD mouse, the full length human mutant huntingtin (fl-*mhtt*) with 97 CAG-CAA mix repeats under control of the human HD promoter gene [22]. This model has not been fully characterized yet and this was the aim of the present studies that are described below.

We first wanted to get a detailed description of possible gait abnormalities in BACHD rats because HD patients have motor impairments such as gait deficits, imbalance, clumsiness and unsteadiness [10]. In this study, we used the Catwalk, an automated video-computer based system that detects and measures a range of spatial and temporal aspects of rodent’s inter-limb coordination during free walking [23]. Also, we wanted to replicate motor imbalance reported in BACHD rats with a Rotarod test [22,24]. The Rotarod has become an essential tool for assessment of motor coordination and balance in rodent models of HD [13,18,25–27]. Finally, we employed an Actimot apparatus to evaluate exploratory locomotor activity of BACHD rats in an open field.

The progressive degeneration of striatal MSN in HD patients may result in deficits in sensorimotor gating inhibition. This ‘gating’ inhibition can be measured in patients by exposing them to startle stimuli. The startle reflex response elicited by either tactile or acoustic stimuli is typically inhibited when it is preceded by a weak prepulse. Interestingly, Swerdlow and colleagues [28] have reported prepulse inhibition (PPI) deficits in HD patients. In animals, startle response and PPI can be derived from the assessment of whole body movement following exposure to auditory or visual stimuli [18,19,29]. Since the PPI test is of particular interest for its ‘translational’ value, we investigated the acoustic startle response (ASR) and prepulse inhibition in BACHD rats.

Neuropsychological assessments found that, in general, cognitive impairments start prior to the emergence of motor deficits, although these are generally diagnosed later [30,31]. Episodic memory – that is, the memory for events which is described as a spatio-temporal record of a subject’s experience - is impaired in HD patients. Some of the most common tests used to measure episodic memory are based on auditory and verbal learning [Rey auditory verbal learning test (RAVLT), California verbal learning test (CVLT), Wechsler memory scale (WMS)], or pattern and spatial recognition. Results from recognition memory performance in patients have led to mixed results and seem to depend on the task that is used. For example, verbal recall memory impairment was reported in the CVLT and WMS tests [32]; whereas performance in spatial recognition memory was intact [31]. A meta-analysis conducted on a computer-based search in presymptomatic, symptomatic and control subjects has revealed deficit in recall-recognition memory in HD [33]. In addition, investigations made in a tgHD rat model have showed spatial and recognition memory deficits at 16 months of age [34]. Although there seems to be a clear impairment in recall-recognition memory in HD patients and tgHD rats, there is a further need to clarify recognition memory performance. Therefore we evaluated the recognition memory in BACHD rats in a novel object recognition task (ORT).

## Materials and Methods

### Ethics statement

The study was carried out in strict accordance with the German animal welfare act and the EU legislation (EU directive 2010/63/EU). The protocol was approved by the local ethics committee *Behörde für Gesundheit und Verbraucherschutz* (BGV, Hamburg).

### Husbandry and genotyping

Wild type (+/+, WT) and transgenic (+/T, TG) male BACHD rats carrying the mutant human huntingtin gene, under the control of the human huntingtin promoter and its regulatory elements were used. The transgene contains 97 CAG-CAA mix repeats, and additional 20 kb upstream and 50 kb downstream sequences ensure stability of the repeat length [22]. Two transgenic males (TG5 line) were supplied from the original BACHD colony of the Universitätsklinikum Tübingen (Tuebingen, Germany) and an in-house breeding colony was established and maintained at EVOTEC AG (Hamburg, Germany) by cross-breeding these males with wild type female rats. BACHD animals were maintained on a Sprague-Dawley background. All the animals at weaning were group-housed 2 to 4 per cage with wood shavings and a filter top. The environment was enriched with a play tunnel and shredded paper. BACHD rats were maintained in climate controlled housing, with a 12-h reversed dark/light cycle (light from 19: 00 to 07:00). Rats had free access to food and water except during experiments.

Ear punches were collected from the litters at weaning in order to determine the rats genotype. Genotyping was performed before and after all the studies using a validated protocol. Briefly, genomic DNA was prepared from ear biopsy tissue using proteinase K digestion, followed by phenol/chloroform extraction (Qiagen DNeasy Tissue kit). Primers flanking the polyQ repeat in exon 1 were designed to recognize whether or not the rat carried at least one copy of the mutation, and were used to PCR amplify the polyQ regions [Q3: 5’ – AGG TCG GTG CAG AGG CTC CTC -3’ and Q5: 5’ – ATG GCG ACC CTG GAA AAG CTG -3’]. Gene status was confirmed in parallel by using designed primers from Tuebingen [exon 1: FW 5’-ATG GCG ACC CTG GAA AAG CTG- 3’ and RV: 5’ -AGG TCG GTG CAG AGG CTC CTC- 3’; exon 67: FW 5’-TGT GAT TAA TTT GGT TGT CAA GTT TT- 3’ and RV: 5’ –AGC TGG AAA CAT CAC CTA CAT AGA CT- 3’]. The PCR product was run on an automated apparatus PTC-200 (Peltier Thermal Gradient Cycler) and the Agilent 2100 Bioanalyser (Agilent technologies) was used to determine the fragments’ size.

### Behavior testing

All the behavioral tests were performed during the dark phase and male BACHD rats were weighed twice per month. Acoustic startle experiments and object recognition tests were undertaken independently on all animals of a cohort at a specific age. For other tests, subgroups of either one or more cohorts were allocated for longitudinal testing. Before each behavioral test, animals were given a 1h minimum habituation period to the testing room.

#### Exploratory behavior

Spontaneous locomotor activity was evaluated with the automated Actimot system (TSE system, Germany). The apparatus consists of a square shape frame equipped with a transparent cage (50 cm^3^) and two pairs of light-beam strips. Each strip is equipped with 32 infrared sensors and their height can be adjusted. Thus, the coordinates of the animal can be determined in three dimensions (X-Y-Z axis). The apparatus was cleaned and dried with a 10% ethanol solution before each use. Each rat was placed in the center of the box and the number of beam breaks recorded during free walking for a 1 hour testing period. We examined and analyzed the total activity (X+ Y beam breaks) directly from the data collected by the system software.

#### Rotarod

Motor coordination and balance was assessed using a rotarod apparatus (Med Associates, Italy). All rats underwent a 3-day training program by which time a steady baseline level of performance was attained. During that period, rats were trained to walk against the motion of a rotating drum at a constant speed of 12 R.P.M (rotations per minute) for a maximum of 2 min. In total, four training trials per day with an interval trial time of one hour were performed. Rats falling off during a training trial were put back on the rotating drum. Following the training days, a one day test of three trials was performed using an accelerating speed levels (4 to 40 R.P.M) mode of the apparatus over 5-min. The apparatus was wiped with a 70% ethanol solution and dried before each trial. The mean latency to fall off the rotarod was recorded and rats remaining on the drum for more than 300 s were removed and their time scored as 300 s.

#### Catwalk system

The gait analysis system Catwalk 7.1 (Noldus IT, the Netherlands) consisted of an enclosed walkway with a glass plate and a speed video recording camera. Gait performance was assessed and recorded using the catwalk analysis software. The glass plate was cleaned and dried with a 70% ethanol solution before each use. On the first day, rats were habituated to the apparatus for 300 s with the goal to cross the walkway. The following day, free runs across the walkway were recorded. From among the correct runs, three runs per animal were selected randomly for analysis. A correct run was defined as one complete (60 cm) crossing of the walkway without interruption. For one crossing, a rat needs a minimum of 4 to 5 step sequences patterns. Runs with step sequence categories related to exploration, Rotate Ra (RF-LF-LF-RH) and Rotate Rb (LF-RF-RH-LH) were not analyzed (RF= right forelimb; RH= right hindlimb; LF= left forelimb; LH= left hindlimb).

The Catwalk parameters are: walking speed (measured as the average of strides in cm/s); the normal step sequences patterns (NSSP, i.e. the order in which the four paws were placed); base-of-support (B.O.S, i.e. distance between two hind or fore paws, as measured perpendicular to the walking direction); stride length (i.e. distance between the placement of a hind or fore limb and the subsequent placement of the same limb); print position (space relation between a fore and a hind paw of the same side in mm); regularity index [RI, i.e. an index for the degree of interlimb coordination during gait, as measured by the NSSP, multiplied by four (number of paws), divided by the number of limb placements, and multiplied by 100%]; stance (i.e. time of contact of the hind or fore limbs with the glass floor); swing (i.e. time that the hind or fore limbs are not in contact with the glass plate); phase of dispersion [i.e. the timing relationships between paw placements. It is expressed as percentage of time of initial contact of one paw (the target) related to the stride length of another paw, the anchor]; max contact (at) (i.e. the point where the breaking phase turns into propulsion phase); intensity (measured as the mean brightness of all pixels of the print at maximum paw contact ranging from 0 to 255 arbitrary units).

Based on the previous study in BACHD mice [16], the hypothesis was to investigate gait abnormalities in the rat model with the same construct. Therefore we focused on the same parameters we identified in the BACHD mice study. Some of the selected parameters were: the normal step pattern sequences, the width between forelimbs placements, stand/propulsion time with their limbs and timing relationships between paw placements. A full description of the apparatus, parameters and analysis is provided by Hamers and colleagues [23].

#### Object Recognition Test (ORT)

Our ORT protocol was similar to a previously described [34]. The apparatus consisted of a circular arena, which was 80 cm in diameter. The floor of the arena and the 35 cm high wall was made of grey polyvinyl chloride.

During testing periods, the experimental room was homogenously illuminated (~ 20 lux). Two objects were placed in a symmetrical position about 20 cm away from the wall. In each trial the objects were placed on the exact same location. Two different sets of objects were used, which had no natural significance or possible association for the rats. The different sets used were: (1) three food cans made of metal painted in blue (diameter 7 cm, height 11 cm) (2), three metal tee caddies cubes (8.5 cm × 8.5 cm × 11 cm, coffee background color) with different shape and size letterings. The objects were secured to the floor to prevent rats from displacing them. On the first day, rats were habituated to the arena without objects for 5 min. In the period preceding the testing, rats were adapted to the arena and all objects 5 min/day for two days.

An ORT testing session (day 4) comprised of two trials (T1 and T2) and the duration of each trial was 3 min. During T1 the arena contained two identical objects. A rat was always placed in the arena facing the wall. After the first exploration period (T1) the rat was returned to its home cage. Subsequently, after a retention interval of 90 min, the rat was put back into the arena containing a familiar (a replicate of the object) and a novel object (T2). The time spent exploring each object during T1 and T2 was recorded with the help of a video tracking system (Ethovision, Noldus). The retention interval time of 90 min was chosen to ensure a reliable recognition memory in WT rat. Exploration of an object was defined as directing the nose towards the object at a distance of no more than 2 cm and/or touching the object with the nose. Sitting on or leaning on an object was not considered as exploratory behavior. In order to obtain reliable results in this task, sufficient exploration time of the objects is critical. The cut-off point for sufficient exploration time was set at 7.5 s total exploration time (both objects) per trial. Since rodents can discriminate between objects based on olfactory cues, the objects were thoroughly cleaned and dried after each trial with a 10% ethanol solution. All combinations and locations (left and right) of the objects were used in a balanced manner in order to prevent potential bias due to preferences for particular locations or objects.

The following variables were calculated:

- (e1) is the amount of the time (in s) spent in exploring both identical objects (a1 and a2) during T1: e1 = a1 + a2
- (e2) is the amount of the time (in s) spent in exploring both the familiar (a3) and new object (b) during T2: e2 = a3 + b
- d1 and d2 correspond to the ability to discriminate between the old and new object during T2: d1 = b − a3, and
  d2 = (b − a3) / e2 (which is d1 corrected for the total exploration time during T2).

A negative d2 indicates a preference for the familiar object; a value of zero indicates no preference, and a positive d2 indicates a preference for the novel object.

#### Acoustic Startle Response (ASR)

The experiments took place in standard “Prime” isolation cabinets from San Diego Instruments (SD Instruments, California). Each ventilated chamber contains a loudspeaker at the top and a cylindrical animal enclosure (10 cm diameter and 20 cm length) made with Plexiglas and mounted on a plastic frame. A piezoelectric accelerometer was mounted under the plastic frame to record and transduce the motion of the tube. All chambers were cleaned with a 70% ethanol solution and dried before each use. Rats were individually placed in the startle enclosure and the resulting movement of the rat was measured during 100 milliseconds (ms) after startle stimulus onset. The response was rectified, amplified and digitized into a computer which calculated the maximal response over the 100 ms period. All rats were habituated for 5-min during which a 70 decibels (dB) background white noise was present. After this habituation period, one of three different experiments was performed similarly to established protocols on independent cohorts [35,36]:. 

### Prepulse Inhibition (PPI)

Rats underwent a test session of 51 trials consisting of: eighteen startle pulse, twenty-four prepulse and nine no stimulus trials where no acoustic pulses were delivered. The startle pulse (P120) consisted of a single 120 dB [A] white noise burst lasting 40 ms. Five pulse-alone (P120) stimuli were presented at the beginning and at the end of the test session. Three types of auditory prepulse at 3, 6, 12 dB above the background noise (70 dB) were presented in random order, followed by a single P120. Each prepulse lasted 20 ms and the prepulse-pulse interval time was 80ms. An average of 16 s (ranging from 10 to 20 s) separated consecutive trials and the total session was approximately 17 min. PPI was calculated as the percent decrease of the ASR in pulse-alone trials compared to the ASR in prepulse-pulse trials [100× ((pulse-alone trial) – (prepulse-pulse trial))/pulse-alone trial].

### Startle Habituation

Rats were subjected to 50 startle trials with an intertrial interval varying between 10 s and 20 s. The startle trial (P120) consisted of a single 120 dB [A] white noise burst lasting 40 ms. Data were analyzed as mean of 10 trials in 5 blocks (BL).

### Startle Threshold

Differences in ASR magnitude for different stimulus intensities (dB) were assessed. Rats received 60 startle trials with varying dB levels ranging from 80 to 120 dB [A] in 10-dB increments, and a no stimulus-free trial (70 dB background noise) per 10 trials. These stimuli lasting 40 ms were randomly assigned to trials within each set of 10 trials. The mean response for all trials of a given dB level, including trials for the 70 dB background white noise for each individual rat was calculated.

#### Statistical Analysis

All data are shown as the mean ± SEM. *Graph Pad* and *InvivoStat* software were used to perform all analyses. Differences between groups were assessed with Student’s t-test or mix ANOVA with repeated measures, with the factor GENOTYPE as between subject and TIME, AGE, INTENSITY or TRIAL as within subject variable. When significance was found, a Bonferroni - post hoc analysis was performed where appropriate. In the ORT test, to evaluate the preference of each genotype, we performed a one sample t-test on the discrimination index. The hypothetical value was set at zero. The significance level was set at 0.05.

## Results

Gross inspection of each BACHD rat prior to the experiments did not reveal any visible differences in phenotype between wild type (WT) and transgenic (TG) animals: all animals appeared healthy. Only male rats were used to avoid any potential effects of changes in the female estrus cycle on behavioral responses. For each test, dedicated cohorts and their size are reported in Table 1.

**Table 1:** Summary of BACHD rat cohort sizes for each behavioral experiment.

**BACHD rats**	**AGE (months)**							
**Behavioral tests**	1	1.5	2	3	4	6	9	12
Weight	(25:15)*		(29:15)	(15:15)	(30:20)	(25:16)	(27:13)	(31:12)
Locomotor Activity	(17:7)	(16:16)	(29:15)	(30:20)	(13:13)	(37:22)	(28:13)	(31:12)
Catwalk						(14:15)		(14:5)
Rotarod	(8:9)		(24:27)	(19:18)	(12:13)	(15:16)	(20:12)	
Object recognition					(15:15)			(14:6)
Prepulse inhibition	(17:17)				(13:13)		(14:15)	(28:12)
Startle habituation						(15:15)		
Startle threshold							(13:15)	

(*) indicates group size (WT: TG).

### Body weight (Fig. 1a)

Both, TG and WT littermate control rats body weight increased over time. A repeated measure ANOVA did not reveal any significant GENOTYPE effect (F(1,276) = 2657.06, P > 0.1), but, as expected, a significant AGE effect was found (F(6,276) = 717.67, P < 0.001). A GENOTYPE x AGE interaction was not found.

**Figure 1:**
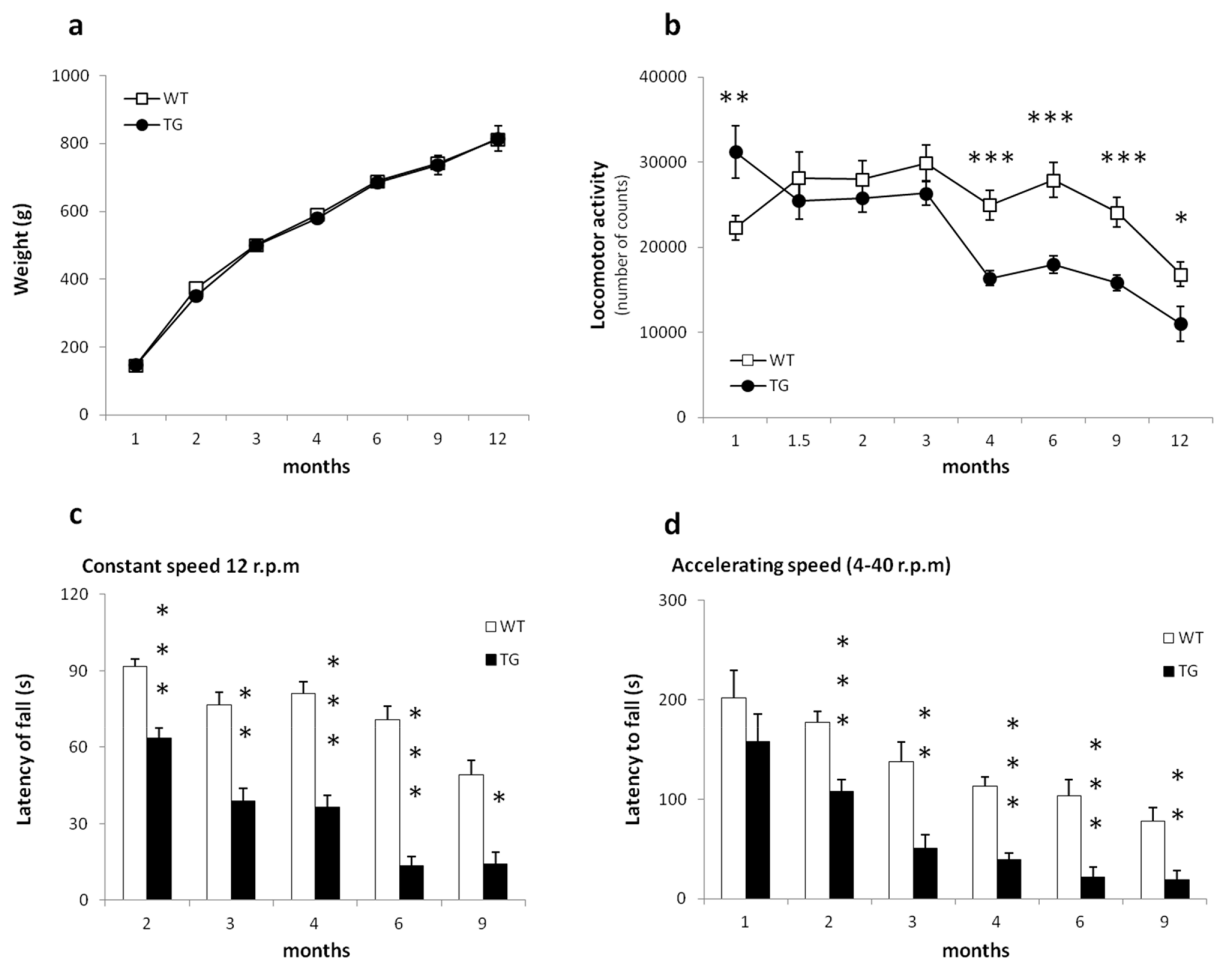
Phenotype. Results are expressed as Mean ± SEM. (a) Body weight. There is no significant difference between TG and WT control rats. (b) Locomotor activity. Compared to WT, TG rats have a higher total activity at one month followed by a lower activity starting at 4 months of age. (c-d) Rotarod. Presented are the latency to fall off the rod during (c) constant speed (12 r.p.m) and (d) accelerating speed (4 - 40 r.p.m). A significant difference between groups was present already at 2 months of age [constant speed (2 months: t= 3.373, P< 0.001; 3 months: t= 3.53, P< 0.01; 4 months: t= 4.798; P< 0.001; 6 months: t=5.433; p< 0.001 and 9 months: t= 2.742, P< 0.05); accelerating speed (1 month: t= 1.066, P> 0. 1; 2 months: t= 4.172, P< 0.001; 3 months: t= 3.549, P< 0.01; 4 months: t= 6.493, p< 0.001; 6 months: t= 4.263, P< 0.001 and 9 months: t= 3.015, P< 0.01)]. Asterisks indicate significant differences between WT and TG rats (*p < 0.05; **p < 0.01 and ***p < 0.001).

### Locomotor activity (Fig. 1b)

In comparison to WT, TG rats showed *hyper*activity at one month of age (t= 2.978, P < 0.01) followed by *hypo*activity starting at 4 months of age (t= 4.415, P < 0.001). This hypoactivity in TG rats persisted till 12 months of age [6 months (t= 3.586, p < 0.001), 9 months (t= 2.784, p < 0.01) and 12 months (t= 2.176, p < 0.05)]. No statistical difference between WT and TG rats was present in 1.5, 2 and 3 months old BACHD rats. A repeated measure ANOVA revealed significant GENOTYPE (F(1,331) = 26.97, P <0.001), AGE (F(7,331) = 10.54, P <0.001) and GENOTYPE x AGE (P < 0.01) effects.

### Rotarod (Figure 1c-d)

Differences in latency to fall off the rotating beam between TG and WT rats were found for training trials at constant speed (Figure 1c, 12 r.p.m) and for testing trials at accelerating speed (Figure 1d, 4 to 40 r.p.m); For technical reasons, results from training at constant speed mode for 1 month old rats cannot be presented. The 12 months old cohort could not be tested because the animals were simply too big to stand on the beam of the rotarod apparatus that we used. Visual inspection of Figure 1(c-d) indicates a progressive decline in rotarod performance for all animals during training at constant speed and test trials at accelerating speed. In fact, TG fell off the rotarod faster than WT rats. This motor coordination deficit and imbalance started at 2 months of age and persisted across time. A repeated measure ANOVA revealed significant GENOTYPE and AGE effects [constant speed: GENOTYPE, F(1,167) = 75.47, P < 0.001 and AGE, F(4,167) = 14.53, P < 0.001; accelerating speed: GENOTYPE, F(1,188) = 58.34, P < 0.001 and AGE, F(5,188) = 17.08, P < 0.001]. No GENOTYPE x AGE interaction was found.

### Catwalk gait analysis system (Figure 2)

We investigated gait deficits in BACHD rats with a Catwalk system. Results from rats of 2 independent cohorts that had made 3 good runs, aged 6 months (WT, n= 6; TG, n= 7) and 12 months (WT, n= 7; TG, n= 3) were analyzed. There was no significant difference in general walking speed at 6 and 12 months. No significant differences in static and dynamic parameters were observed between WT and TG rats at 6 months (data not shown). However, at 12 months, TG rats exhibited a shorter stride length (front limbs, t= 2.194 and p<0.05; hind limbs, t= 2.355 and P< 0.05), shorter stand (the duration contact with the glass plate; front limbs, t= 2.107 and p<0.05; hind limbs, t= 2.690 and P< 0.05) and shorter front limbs swing (t= 2.871 and p< 0.01). There was a statistical trend for a difference in hind limb swing (the duration of no contact with the glass plate; t= 1.819 and p = 0.079). No significant differences were found for the other parameters (data not shown).

**Figure 2:**
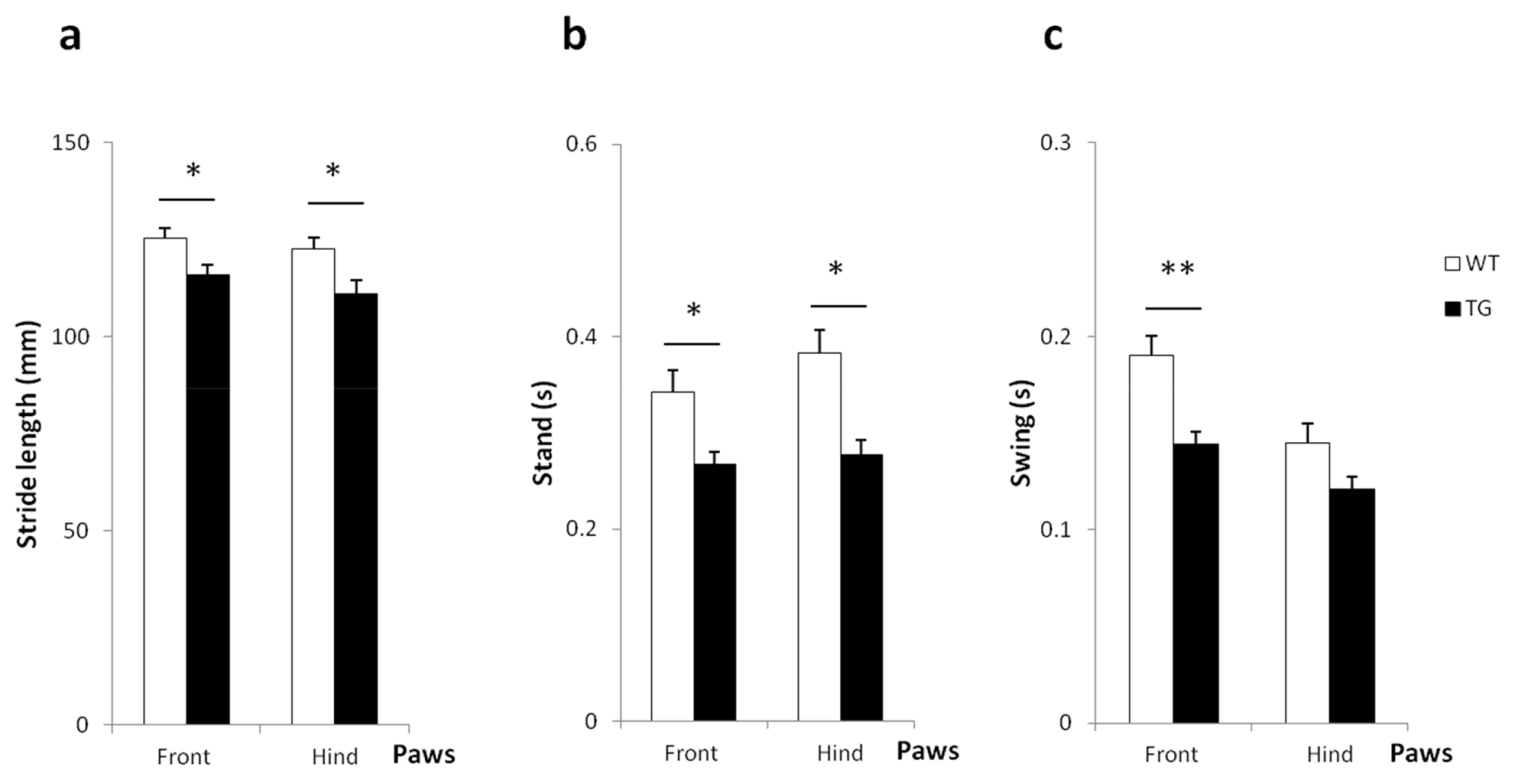
Catwalk gait analysis in 12 months old BACHD rats. Results are expressed as Mean ± SEM. There was no significant difference between TG and WT control rats in walking speed (data not shown). (a) Static parameters: Stride length. Compared to WT, TG rats had a significant shorter stride length for both front and hind paws during walking. (b-c) Dynamic parameters: presented are the Stand (c) and the Swing (d). TG rats had a significant shorter time of stand (or Stance) for front and hind paws and a shorter time in front swing than WT rats. Asterisks indicate significant differences between WT and TG rats (*p < 0.05 and **p < 0.01).

### Object Recognition test (ORT) (Figure 3)

**Figure 3:**
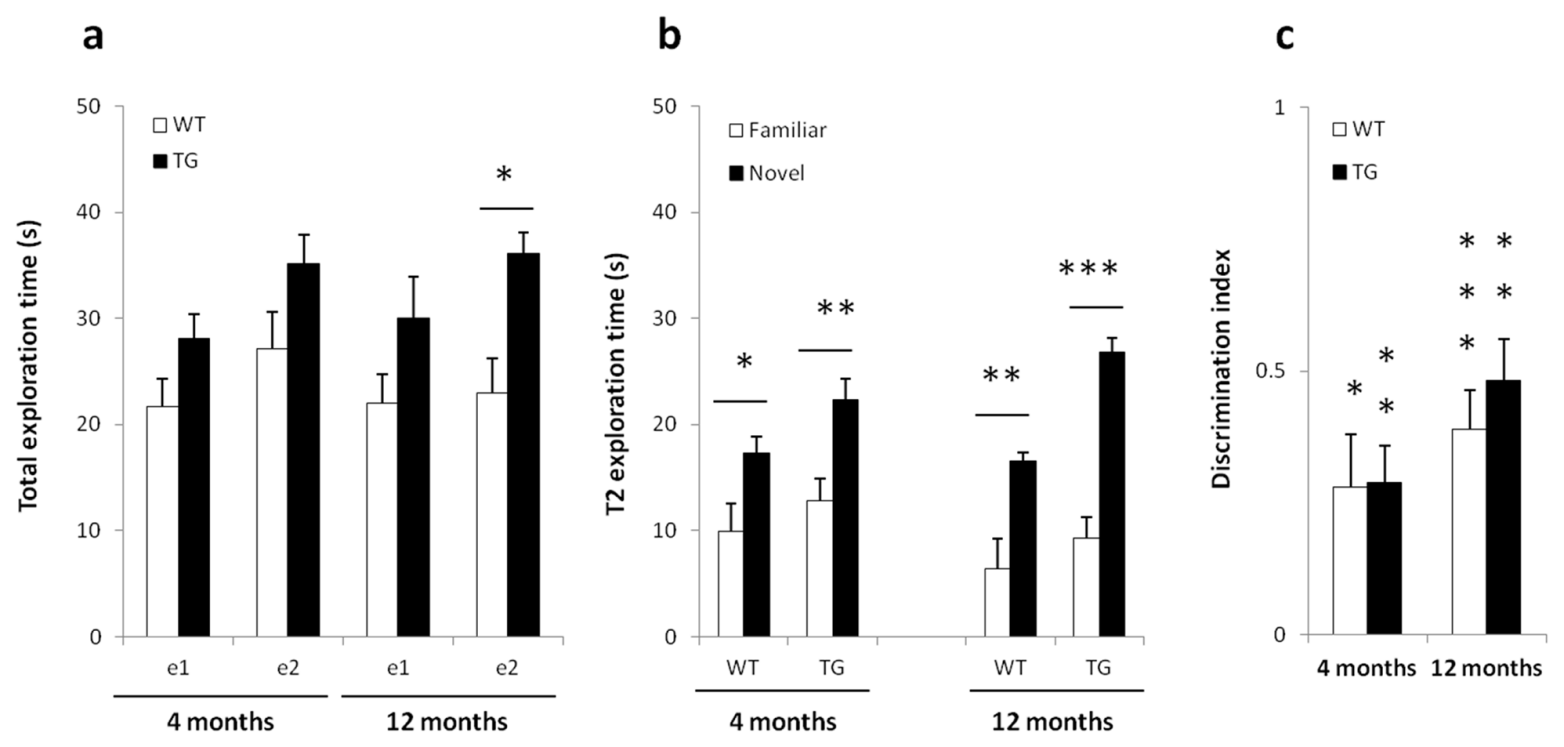
Object Recognition Test (ORT). Results are expressed as Mean ± SEM. Three WT rats of the 4 months old group and one WT rat from the 12 months old group were excluded from the analysis as they did not explore the objects (a) Exploration time (e1) and (e2) during T1 and T2 respectively. TG rats showed a significantly higher exploration time during e2 at 12 months of age. (b) Recognition testing during T2: 4 months and 12 months old BACHD rats had a significantly higher exploration time to the novel object than the familiar object. (c) The discrimination index (d2) for both groups are above zero and were significant at each age. Asterisks indicate statistical significance (*p < 0.05; **p < 0.01 and ***p < 0.001).

Three WT rats of the 4 months old group and one WT rat from the 12 months old group were excluded from the analysis as they did not explore the objects. Visual inspection of Figure 3a indicates that TG rats had a higher total exploration time (e2). Indeed, during T1 and T2, significant GENOTYPE and TIME effects were found in four months old [2-way ANOVA: GENOTYPE, F(1,25) = 4.75, P< 0.05; TIME, F(1,25) = 9.45, P< 0.01] and 12 months old rats (GENOTYPE, F(1,18) = 4.92, P< 0.05; TIME, F(1,18) = 4.87, P< 0.05). Bonferroni post hoc testing revealed significant differences only for (e2) exploration time in 12 months old rats (t = 2.612, P < 0.05). No interaction between GENOTYPE and TIME was found.

Analysis of the recognition performance during T2 (Figure 3b) revealed a TIME effect at 4 months of age [2-way ANOVA: GENOTYPE, F(1,25) = 3.482, P> 0.05; TIME, F(1,25) = 16.62, p< 0.001] whereas a GENOTYPE and TIME effects were found at 12 months of age (GENOTYPE, F(1,18) = 6.589, P< 0.05; TIME, F(1,18) = 39.34, p< 0.001). In all, WT and TG had a significantly higher exploration time for novel objects than familiar objects at both ages (4 months: WT, t = 2.389, P< 0.05 and TG, t = 3.444, P< 0.01; 12 months: WT, t = 4.218, P< 0.01 and TG, t = 4.735, P< 0.001).

The relative discrimination index (d2) was positive in both age groups (Figure 3c). A one sample t-test revealed a statistically significant difference from zero for both 4 months old (WT, t = 2.8, P< 0.05; TG, t = 4.1, P< 0.01) and 12 months old (WT, t = 5.38, P< 0,001; TG, t = 6.18, P< 0.01) rats.

### Acoustic Startle Response (ASR) (Figure 4)

We evaluated sensorimotor gating in different BACHD rat cohorts. Prepulse inhibition (PPI) was assessed in 1, 4, 9 and 12 months old rats, whereas startle habituation and startle threshold were evaluated in 6 and 9 months old rats, respectively. A first look to the results yields very little with large standard errors in PPI and startle threshold (Data S1). Given the fact that we have rigorously executed the experiments with fairly good large size of animals per test and time points, possible confounding factors could be attributed to outliers. Therefore, we performed a Grubb’s test analysis and as a result removed the outliers, which indeed significantly reduced the variability of the groups.

**Figure 4:**
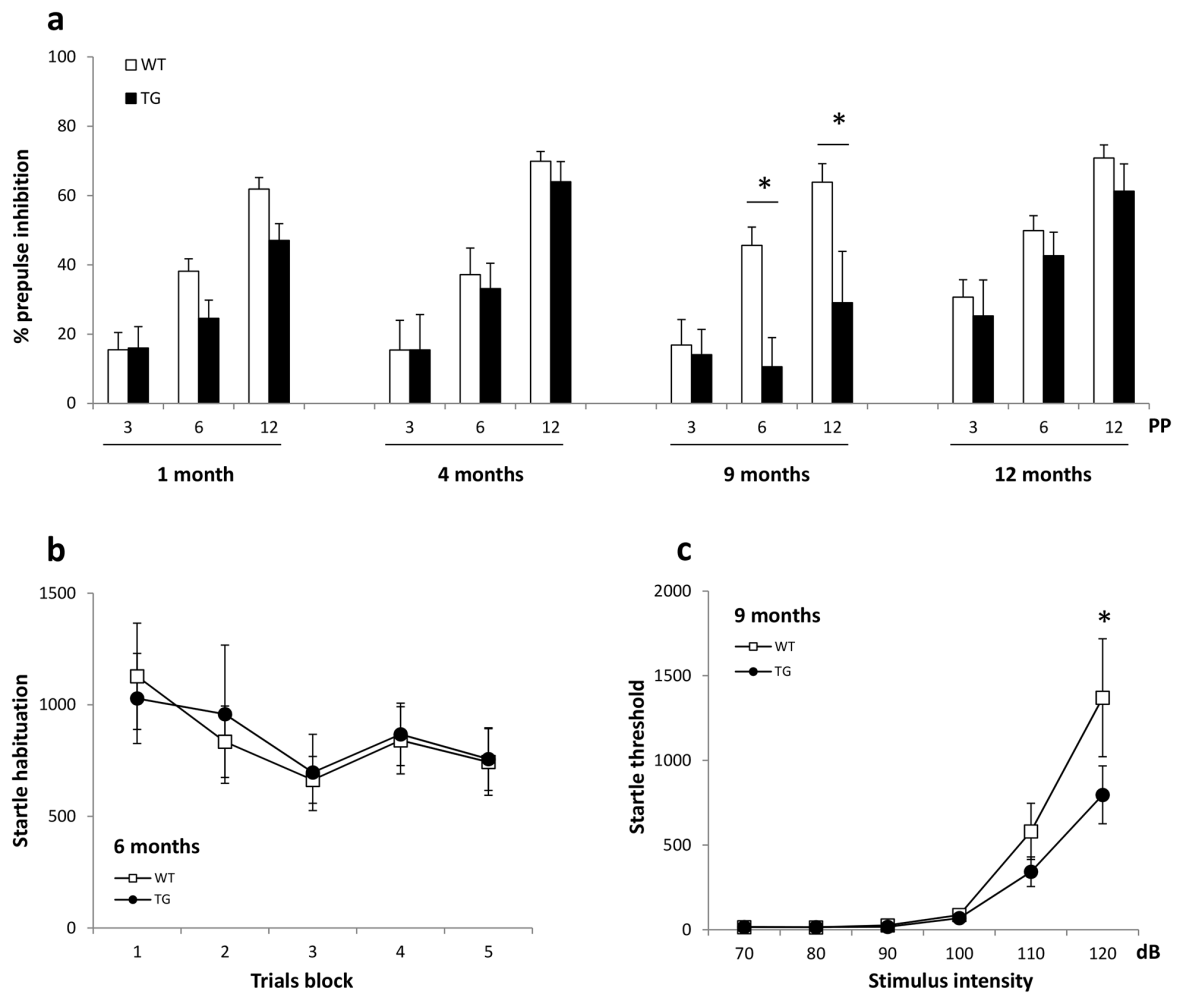
Startle testing. Results are expressed as Mean ± SEM. (a) Prepulse inhibition. A 2 way-ANOVA revealed a GENOTYPE difference in 9 months old BACHD rats especially at PP 6 and PP 12; however any significant differences were found in 1, 4 and 12 months old rats. (b) Startle habituation amplitude in 6 months old rats. Each trial consisted of 10 blocks of 120 dB startle stimuli. WT and TG rats presented a normal startle habituation. (c) Startle threshold. Amplitude to varying startling stimulus intensities in 9 months old rats. WT and TG response amplitude increased with higher stimulus intensities. No GENOTYPE effect was observed. However, a significant difference was detected at 120 dB. Asterisks indicate statistical significance in BACHD rats (*p < 0.05).

### Prepulse Inhibition (PPI) (Figure 4a)

For the PPI experiments, prepulse intensities (PP) of 3, 6, 12 dB above background were used. One month old (17WT:16TG), 4 months old (12WT:13TG), 9 months old (13WT:14TG) and 12 months old (25WT:11TG) BACHD rats data were analyzed. A 2-way ANOVA analysis with GENOTYPE as a between subjects factor and TRIAL (PP3, PP6, PP12) as a within subjects factor revealed a significant GENOTYPE and TRIAL effect in 9 months old rats [GENOTYPE: (F(2,25) = 5.42, P< 0.05; TRIAL: F(2,50) = 12.96, P< 0.0001) with PP6, t = 2.78, P< 0.05 and PP12, t= 2.76, P< 0.05 Bonferroni post hoc test)]. An interaction between GENOTYPE and TRIAL was detected (F(2,50)= 4.589, P< 0.05). No significant GENOTYPE effect was found at 1, 4 and 12 months of age; however a significant TRIAL effect was detected in all groups [(1 month, F(2,62)= 65.02, P< 0.0001; 4 months, F(2,46)= 53.14, P< 0.0001 and 12 months, F(2,68)= 41.27, P< 0.0001)]. There was no interaction between GENOTYPE and TRIAL. A close inspection of the data for the 1 month old rats suggests a difference between WT and TG rats at PP6 and PP12. A post hoc analysis of 1 month old rats revealed a trend to significance at PP12 (PP6, t = 2.026, P = 0.136; PP12, t = 2.206 and P = 0.089). This suggests that 1 month old transgenic rats have mildly impaired sensorimotor gating. Together with 4 and 12 months old rats from Figure 4 data, the deficits were too subtle to reveal a significant, overall, genotype difference between WT and TG rats at any of the other prepulse intensities following post hoc analysis. Finally, analysis of startle amplitude on P120 alone trials in BACHD rats did not show differences between groups (data not shown).

### Startle Habituation (Fig. 4b)

To further evaluate startle habituation in BACHD rats, six months old rats were presented 50 trials of a 120 dB (P120) startle stimulus. The results are presented in 5 blocks of 10 trials. Visual inspection of Figure 4b suggests a general decrease in startle amplitude. This was confirmed by a 2-way ANOVA with a significant TRIAL effect (F(4,112) = 3.36, P< 0.05). However, no statistically significant GENOTYPE effect or GENOTYPE and TRIAL interaction was found.

### Startle Threshold (Figure 4c)

We evaluated 9 months old BACHD rat’s response magnitude to different stimulus intensities; five startle intensities (80dB to 120 dB), 10 trials each, were presented. Ten WT and 12 TG BACHD rats’ data were analyzed. WT and TG startle amplitude increased with the startle stimulus intensity. A 2-way ANOVA analysis revealed a significant INTENSITY effect at 120 dB [INTENSITY, F(5,100) = 26.80, P< 0.0001 and 120 dB, t = 2.965 and P < 0.05]. However, there was no over-all GENOTYPE effect and no interaction between GENOTYPE and INTENSITY was found.

## Discussion

We evaluated transgenic BACHD rats and wild type littermate control in a series of standard behavioral tests assessing motor, sensory-motor and cognitive function. TG rats showed a clear motor coordination imbalance on a Rotarod, as well as gait coordination deficits in both static and dynamic free walking sequences as measured with a catwalk system. These data demonstrate a progressive motor impairment over time as seen in patients. Rats showed intact recognition memory as measured in an object recognition test. A clear deficit in sensory-motor paradigms (startle habituation, and startle threshold) was absent, although a subtle impairment in PPI was found. Whereas we confirmed the motor phenotype as described previously [22], we expected a more profound cognitive and sensorimotor phenotype. Additional studies will be needed to provide further insights into the validity of the BACHD rat model for HD and how this model compares with other rodent models for HD.

### Motor Behavior

The first clinical reports on HD predominantly addressed motor symptoms, including progressive involuntary movements named “chorea” [37]. Therefore, we started first with the assessment of motor behavior in our comprehensive behavioral phenotyping approach of BACHD rats.

A first report on ambulatory activity in BACHD rats found hypoactivity at 3 months of age [22]. In the present study, TG rats showed *hyper*activity in an open field environment at 1 month followed by a progressive and long-lasting *hypo*activity starting at 4 months. Similar observations have been reported in YAC 128 mice with a hyperkinetic phenotype at 3 months followed by hypokinetic phenotype at 6 months of age [38]. However, results from other rodent models for HD have been variable. For example, *hyper*activity was found in transgenic tgHD rats carrying 51 CAG-repeats, with higher exploratory distance travelled in an open field test at 6, 7, 8 and 10 months. A progressive *hypo*activity phenotype was found in transgenic BACHD mice, starting at 7 months [18,39].

The *hype*ractivity detected in BACHD rats at an early age (4 weeks) was only expressed for a very short time window as it had disappeared at 6 weeks. Yu-Taeger and colleagues have probably missed this phenotype in BACHD rats as they started testing at 3 months of age. However, the robustness of this phenotype needs to be addressed in follow-up studies with a higher number of animals (there were only 7 rats in the one month old transgenic cohort). Nevertheless, it is encouraging that the biphasic activity pattern in our rats appears to mimic more closely some of the clinical neuropsychiatric symptoms reported in pre-manifest and manifest HD patients [40,41].

Measurement of motor coordination and balance on a rotarod showed a clear deficit in TG rats as they have a shorter latency to fall off the rotating rod during constant speed training trials and during accelerating speed trials. It is interesting to note that the performance of WT rats on the rotarod declines over time. Indeed, weight gain in rats can impact rotarod performance; however the results demonstrate that the difference we observed between WT and TG BACHD rats is unlikely to be influenced by animal’s weight. The same observations were made by Yu-Taeger and colleagues [22], with TG rats having difficulties maintaining balance on the rod at higher rotation speeds. The progressive imbalance in rats persists over time and is consistent with data obtained from BACHD mice and tgHD rats [13,16–19,26,42]. Taken together, these data prove the reliability of using the Rotarod test for motor coordination assessment across laboratories.

Gait abnormalities were found in both static and dynamic parameters during Catwalk testing in 12 months old rats. TG rats had a shorter stride length and decreased stand duration of front and hind paws. A decrease in swing duration was observed for the front paws. Although velocity can significantly influence catwalk gait parameters [43], the deficits in BACHD rats are unlikely to be confounded by this parameter as TG and WT rats did not differ in walking speed. Footprints of TG rats have been previously investigated for gait abnormality at 14 months of age. Shorter steps for limbs, increased stride width and reduced overlap between forelimbs and hindlimbs placement were found [22]. In accordance with our findings, similar results have been reported in other HD rodent models. For example, tgHD rats also showed decreases in stand and swing duration in a Catwalk test [44]. R6/2 transgenic mice displayed a significantly shorter stride length by 8-9 weeks of age in an ink-footprint test, and shorter stance time for front and hind limbs at 17 weeks in a Digigait system during treadmill locomotion [25,45]. Finally, HD patients show gait abnormalities like mean decrease in velocity, stride length and cadence [46].

Based on the early and profound coordination deficits in the Rotarod, we did not expect the late occurrence of relative mild gait abnormalities in TG rats. However, consistent with the late onset in BACHD rats, we found gait abnormalities at an advanced age (10 months) in BACHD mice such as: differences in the NSSP cruciate and alternate, larger distance between forelimbs placements, shorter stand/propulsion time with their hindlimbs and timing relationships between paw placements [16]. However, none of these deficits were found in 12 months old BACHD rats in the present study. Methodological confounds cannot be excluded but seem unlikely as the mice and rats were tested in the same laboratory, by the same experimenter, using the same equipment. This illustrates that the disease progression in 2 different species with a same construct might be different. Although gait deficits appear to be present at 14 months of age with the footprints test, it would be worthwhile investigating if major deficits in Catwalk performance are present in BACHD rats that are older than 12 months*.*

### Object recognition

Recognition memory was investigated in BACHD rats in an ORT task and intact object memory was found in 4 and 12 months old rats. A close look at the recognition performance of 12 months old rats during T2 suggests a better cognitive performance in TG rats. Unfortunately, the design of the experiments (i.e. using a relatively short inter trial interval) was aimed at inducing a robust object recognition in WT rats. One way to investigate the hypothesis that TG rats actually have superior performance would be to increase the inter trial time between T1 and T2 and observe if WT rats performance decrease faster. This would, however, be beyond the scope of this study, which was signed to investigate only whether TG rats have reduced recognition memory. Given that both transgenic and control groups exhibit a significant positive discrimination index, we can conclude that their recognition memory is intact. Our results contrast with findings in another rat model for HD. tgHD rats show deficits in ORT and OLT (object location test) at 16 months of age [34] and cognitive deficits have been reported as early as 9 months of age with different tasks assessing visual-spatial learning and memory processing [47–49]. It cannot be ruled out that we might have missed a recognition memory deficit as BACHD rats show no clear *htt* aggregates or neurodegeneration before 12 months of age [22]. Therefore, we assume that in contrast to motor behavior, the circuitry involved in ORT may become dysfunctional only if *htt* aggregates have formed. Further studies need to be done in animals and humans to better understand if and how object memory is impaired in HD. Object memory seems to be impaired in HD patients. In a pattern recognition task (a task similar to the rat ORT), subjects had to remember and touch the abstract patterns they were shown during training and that were paired with a novel pattern during testing. Early HD patients and clinically symptomatic subjects performed significantly worse than control subjects [50,51], whereas in at-risk gene carriers, no difference in recognition memory was found [31]. Irrespectively, this is the first study to report intact object memory in BACHD rat and further studies in older cohorts may shed further light on a potential recognition memory deficit.

### Acoustic Startle Response

Nine months old BACHD transgenic rats had a significant prepulse inhibition deficit at 6 and 12 dB prepulse (PP) intensities. Closer inspection of 1 month old rats’ data suggests subtle deficits at PP 6 and 12 dB. However, no genotype differences were found in 1, 4 and 12 months old rats. Likewise, no statistical difference was detected in startle responding and startle habituation.

PPI deficits have been demonstrated in BACHD mice and YAC 128 mice models for HD at later ages [18,19,52]. Impaired PPI has been shown in the neurotoxic rat model after systemic administration of 3-Nitropropionic acid [53,54]. Also, PPI has been extensively investigated in several neuropsychiatric disorders [55–57]. In HD patients, only Swerdlow and colleagues [28] have reported reduced PPI in both acoustic and tactile startle reflex whereas no impairment was observed in startle amplitude, habituation or prepulse latency facilitation paradigms. In view of 1) the correlations between PPI impairments and degenerative changes in the striatum of animal models and HD patients; and 2) the absence of clear neurodegeneration or striatal *htt* aggregates in caudate putamen (CPu) before 12 months of age [22], it cannot be ruled out that a PPI deficit develops after 12 months of age. Unfortunately, we could not test older rats since they were too big for the standard PPI rat enclosures. Although this technical issue is certainly not insurmountable, an interesting alternative would be to test PPI in a non-human primate model for HD [58]. Testing in a different species would help to shed more light on the robustness of a sensorimotor gating deficit in HD models.

## Conclusion

In the present time course study, we investigated motor, sensorimotor and cognitive symptoms in BACHD rats. Transgenic rats showed motor coordination imbalance on a Rotarod and subtle gait deficits in a Catwalk system. These rats had an intact object recognition memory and a subtle deficit in prepulse inhibition of acoustic startle. In contrast to the symptoms progression in patients, BACHD rats may not show object memory impairment until after motor deficits occurred. Further assessment of other cognitive functions, such as reversal learning and associative memory, may shed further light on the comparative time courses for the emergence of motor and cognitive deficits.

If deficits in sensorimotor and cognition functions are linked to *htt* aggregation, it is possible that our BACHD rats were too young (12 months) to show robust deficits. Yu-Taeger and colleagues [22] revealed that nuclear accumulation of N-terminal *htt* appeared in cortex after 9 months of age and few aggregates were present in the dorsolateral caudate putamen from 12 months old rats and increases thereafter. However, the relationship between mutant huntingtin (m*htt*) aggregation and MSN loss, motor and cognitive deficits in BACHD rodent models for HD appears complex. Sometimes symptoms occur before the neuropathology like in BACHD mice, where few m*htt* aggregates are present at 12 months of age in cortical and striatal regions [13,17,19,20]. Further studies need to be performed to better understand the molecular and cellular mechanisms underpinning motor and cognitive symptoms in BACHD rats.

## Supporting Information

Data S1 Startle testing. Results are expressed as Mean ± SEM. (a) Prepulse inhibition and (b) Startle threshold amplitude in BACHD rats. 2 way ANOVA: Prepulse inhibition [GENOTYPE: (1 month, F (1,32) = 3.84, P = 0.0587; 4 months, F (1,24)= 0.376, P= 0.545; 9 months F (1,27)= 1.447, P= 0.23 and 12 months, F (1,38)= 0.311, P= 0.58); TRIAL: (1 month, F (2,64) = 68.22, P = 0.0492 with PP 12, t= 2.438 P= 0.0498 ; 4 months, F (2,48)= 10.74, P= 0.0001, 9 months F (2,54)= 13.22, P> 0.0001 and 12 months, F (2,76)= 16.25, P> 0.0001) ]; Startle threshold [9 months, GENOTYPE: F (1.26) = 2.109, P= 0.158); INTENSITY: F(5,130)= 23.73, P> 0.0001 with 120 dB, t = 3.473 and P = 0.004]. The general observation of data indicated that no statistical differences in over all GENOTYPE might potentially be due to some outliers. The results without outliers are presented in figure 4.
(TIF)
