# Supplementary figures and images for: Assessment of Motor Function, Sensory Motor Gating and Recognition Memory in a Novel BACHD Transgenic Rat Model for Huntington Disease
Source: PLoS One. 2013 Jul 11;8(7):e68584. doi: 10.1371/journal.pone.0068584 (PMC3708912; doi:10.1371/journal.pone.0068584)

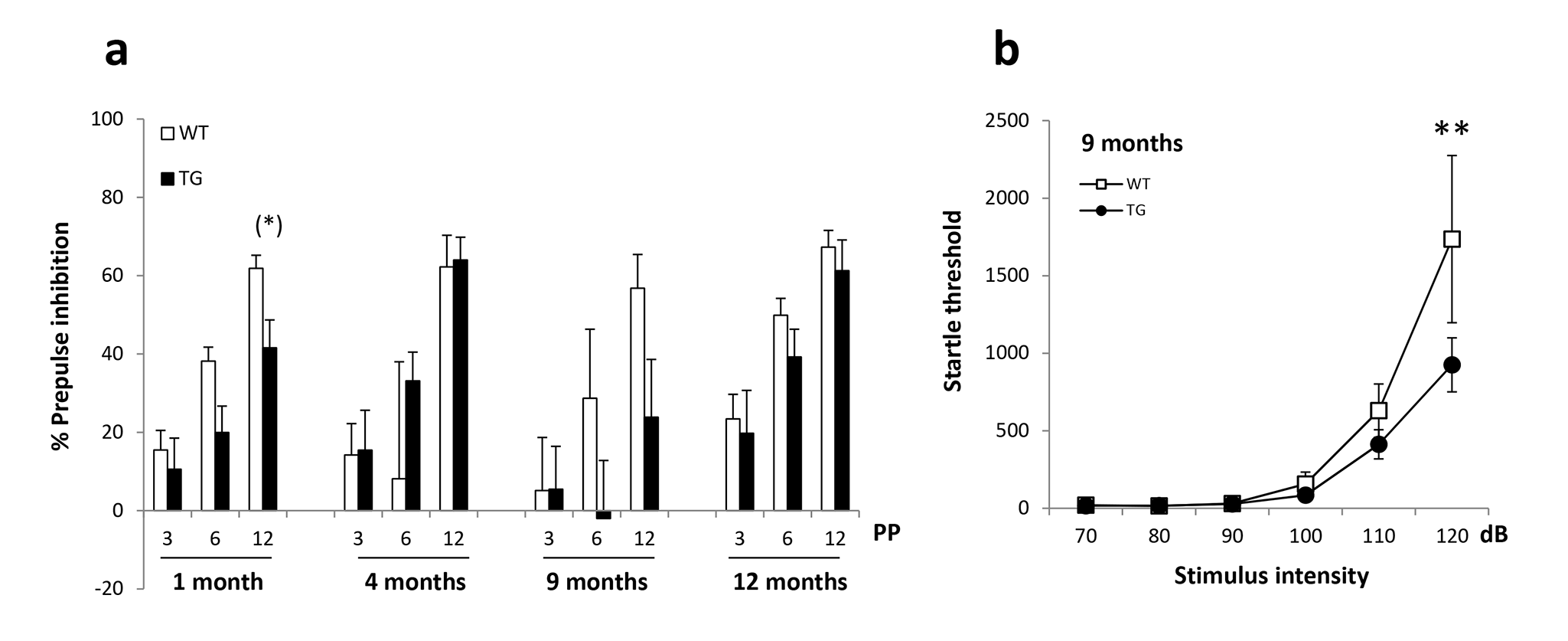

Supplement: Data S1 — Results are expressed as Mean ± SEM. (a) Prepulse inhibition and (b) Startle threshold amplitude in BACHD rats. 2 way ANOVA: Prepulse inhibition [GENOTYPE: (1 month, F (1,32) = 3.84, P = 0.0587; 4 months, F (1,24)= 0.376, P= 0.545; 9 months F (1,27)= 1.447, P= 0.23 and 12 months, F (1,38)= 0.311, P= 0.58); TRIAL: (1 month, F (2,64) = 68.22, P = 0.0492 with PP 12, t= 2.438 P= 0.0498 ; 4 months, F (2,48)= 10.74, P= 0.0001, 9 months F (2,54)= 13.22, P> 0.0001 and 12 months, F (2,76)= 16.25, P> 0.0001) ]; Startle threshold [9 months, GENOTYPE: F (1.26) = 2.109, P= 0.158); INTENSITY: F(5,130)= 23.73, P> 0.0001 with 120 dB, t = 3.473 and P = 0.004]. The general observation of data indicated that no statistical differences in over all GENOTYPE might potentially be due to some outliers. The results without outliers are presented in figure 4. (TIF) [file pone.0068584.s001.tif]
